# Deciphering the Phytochemical Potential of Hemp Hairy Roots: A Promising Source of Cannabisins and Triterpenes as Bioactive Compounds

**DOI:** 10.3390/molecules29235792

**Published:** 2024-12-07

**Authors:** Naomi Kaminsky, Jane Hubert, Cédric Guerin, Malak Mazlani, Alexis Kotland, Victor Pozzobon, Blandine Marant, Héloïse Mailhac, Stéphane Poigny

**Affiliations:** 1 Sativa Towards Health Biotechnologies (STH BIOTECH), 91000 Evry-Courcouronnes, Franceh.mailhac@sth-biotech.com (H.M.); 2 NatExplore, 51140 Prouilly, France; 3 Université Paris-Saclay, CentraleSupélec, Laboratoire de Génie des Procédés et Matériaux, Centre Européen de Biotechnologie et de Bioéconomie (CEBB), 3 rue des Rouges Terres, 51110 Pomacle, France; 4 INRAE, RIBP USC 1488, Université de Reims Champagne Ardenne, 51100 Reims, France; 5 Mibelle Group Biochemistry, Mibelle AG, 5033 Buchs, Switzerland

**Keywords:** *Cannabis sativa*, hairy root, phytochemistry, cannabisins, triterpenes, bioactivity, antioxidant, antifungal

## Abstract

*Cannabis sativa* L., specifically hemp, is a traditional herbaceous plant with industrial and medicinal uses. While much research has focused on cannabinoids and terpenes, the potential of hemp roots is less explored due to bioproduction challenges. Still, this material is rich in bioactive compounds and demonstrates promising anti-inflammatory, antimicrobial, and antioxidant properties. Biotechnological methods, such as hairy root cultures, enable the efficient production of specialized metabolites while avoiding the issues of outdoors cultures. Despite these benefits, the chemical diversity understanding of hemp hairy roots remains limited. In this study, we conducted an extensive NMR and LC/MS chemical profiling of hemp hairy roots to determine their chemical composition, revealing the presence of cannabisins for the first time. We then investigated the accumulation of cannabisins and triterpenes in both hemp hairy roots and hemp aeroponic roots. Our findings reveal that hairy roots produce 12 times more cannabisins and 6 times more triterpenes than aeroponic roots, respectively, in addition to yielding 3 times more biomass in bioreactors. Preliminary bioassays also suggest antioxidant and antifungal properties. This research underscores the potential of hemp hairy roots as a valuable source of specialized metabolites and calls for further exploration into their bioactive compounds and applications.

## 1. Introduction

*Cannabis sativa* L., including hemp varieties, is an annual, herbaceous, dicotyledonous plant from the *Cannabinaceae* family, and it has been cultivated since Neolithic times. Industrial hemp has been mainly exploited for its fibers in textile, paper, and construction [1], as well as for its lipids and proteins-enriched seeds in nutrition [2]. *C. sativa* biosynthesizes various specialized metabolites, mainly in glandular trichomes, where cannabinoids and terpenes are accumulated [3,4]. Although research has mainly focused on metabolites from glandular trichomes for their therapeutic properties [5], other parts of the hemp plant are also valuable sources of specialized metabolites. Indeed, monoterpenes and diterpenes have been reported in inflorescences, while sesquiterpenes, triterpenes, alkaloids, and phytosterols have been described in roots and flavonoids in the leaves [6].

Hemp roots are used in traditional medicine for their anti-inflammatory and pain-relieving properties, among others [7,8,9,10]. For instance, hemp root extract demonstrated the inhibition of interleukin-1β (IL-1β) production in THP-1 cells, which are a human monocyte cell line [7]. The phytochemical content of these hemp root extracts accounts for their biological activity. Indeed, several studies have highlighted a diversity of specialized metabolites in hemp roots, including monoterpenes such as carvone and dihydrocarvone [6,8]; triterpenes such as friedelin and epifriedelanol [6,11,12,13]; sterols such as β-sitosterol, campesterol, and sigmasterol [6,11,13]; alkaloids such as pyrrolidine, piperidine, cannabisativine, and anhydrocannabisativine [14,15,16]; and lignanamides such as *p*-coumaroyltyramine [17,18,19]. Despite hemp roots’ promising content, several limitations exist to their exploitation. First, root growth is slow, and this biomass is complex to harvest in soil. Second, hemp roots cultivated in soil may also absorb pollutants, including heavy metals and other soil wastes [20], which could restrict their use for health and well-being applications.

Alternative cultivation methods, such as hydroponic and aeroponic cultures, were developed to avoid these drawbacks while preserving the production of active molecules. These methods can induce the accumulation of specialized metabolites in roots, without the limitations of polluted soils [21,22,23]. Aeroponic cultures can demonstrate increasing biomass and secondary metabolite production. For instance, aeroponic roots of *Coffea arabica* showed a significant increase in secondary metabolites like caffeine and theobromine, as well as greater biomass yield in both the leaves and roots, compared to traditional soil-based systems [24]. In another study, *C. sativa* aeroponic roots showed fewer accumulation of triterpenes, such as friedelin and epifriedelanol, compared to soil-grown roots; still, the overall yield was higher due to significantly higher biomass production [12,25]. However, it is important to consider the environmental impact of using aeroponic systems. For instance, in caffeine production, electricity consumption is higher in aeroponic cultures compared to soil cultivation [24].

Over the last decades, developing in vitro cultures has provided new opportunities to produce phyto-molecules by biotechnologies, including hairy root cultures. This method involves plant transfection with *Rhizobium rhizogenes*, a Gram-negative soil bacterium inducing the so-called “hairy root syndrome” that naturally occurs in the infected host plant. While *R. rhizogenes* transfects a plant, its T-DNA region (composed of *rol* A, B, C, and D) is randomly and stably incorporated into the plant genome, resulting in an excessive proliferation of fast-growing root tissues in hormone-free media, without any light needs [26,27]. This root tissue benefits from genetic stability over time [28,29] and can be grown in a sterile controlled environment such as a bioreactor. This result in optimized and consistent productions with fewer variations in biomass and metabolite production [30,31]. Moreover, hairy roots enable the growth of biomass from difficult-to-cultivate plants or protected endemic species [32]. These elements support the use of hairy roots as a biofactory for producing plant secondary metabolites [33,34]. Furthermore, they can accumulate metabolites not present in their mother plants using metabolic engineering [35,36,37]. Previous studies reported that hemp hairy roots accumulate atropine, choline [38], friedelin, and epifriedelanol [39,40]. Farag et al. (2015) proposed an in vitro method to obtain adventitious root tissues from dedifferentiated calli, which produced minor cannabinoids [41]. Despite the increasing use of hairy roots as a source of plant-specialized metabolites, the chemical diversity of hemp hairy roots remains uncharacterized.

In this study, we established a process to obtain large amounts of hemp hairy roots and investigated their chemical composition. Through Nuclear Magnetic Resonance (NMR) and high-resolution Liquid Chromatography coupled with Mass Spectrometry (LC-MS) analyses, we revealed a range of fascinating molecules in hemp roots, such as cannabisins and triterpenes. These molecules were then further quantified in hemp hairy roots and aeroponic roots using Gas Chromatography–Flame Ionization Detection (GC-FID) and Ultra-High-Performance Liquid Chromatography coupled with Quadrupole Time-of-Flight Mass Spectrometry (UHPLC-QtoF), respectively. Finally, antifungal and antioxidant activities were assayed in both extracts. We demonstrated that cultivating hemp hairy roots in a bioreactor results in higher biomass production and significantly higher accumulation of cannabisins and triterpenes compared to aeroponic culture. Hemp hairy root extract also demonstrates more efficiency in bioassays. These results highlight the relevance of using hemp hairy root cultures to produce highly bioactive cannabis metabolites.

## 2. Results and Discussion

### 2.1. Hemp Hairy Roots (HHRs) and Hemp Aeroponic Roots (HARs)

**Figure 1:**
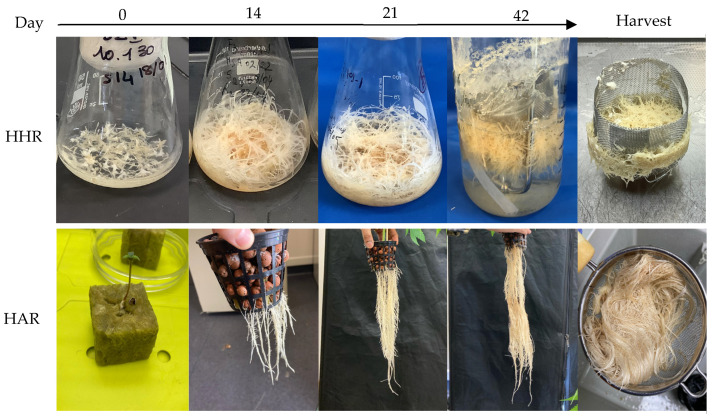
HHR and HAR development from 0 to 42 days of culture and harvested biomass. In vitro HHRs were grown in Erlenmeyer for 21 days, transferred to a 1 L-bioreactor (*n* = 3), and further cultivated until the harvest at 42 days. HARs were grown from 10-day plantlets (*n* = 3) and transferred to a semi-controlled indoor aeroponic system until the harvest on day 42.

HHRs and HARs were grown to observe the difference in biomass accumulation between both techniques. Figure 1 illustrates different stages of biomass development over 42 days. HHRs were cultivated in vitro and are characterized by their short, white, and thick appearance, whereas HARs exhibit a longer and smoother morphology (Figure 1).

The accumulated fresh and dry biomass are detailed in Table 1. Over 42 days of culture, 171.93 g and 9.54 g of fresh and dry biomass were obtained using HHRs compared to 81.23 g and 3.21 g of fresh and dry biomass obtained using HARs (Table 1). Total HHR amounts of 57.31 g Fresh Weight (FW) and 3.18 g Dry Weight (DW) were obtained on average, corresponding to 114.62 g FW/L and 1.59 g DW/L of biomass yield. These results indicate that HHRs were twice as efficient to develop biomass, which in vitro hairy root cultures provided better control and repeatability in our condition.

In our study, the in vitro cultivation of HHRs was less water- and energy-consuming compared to the HAR cultures. For instance, the HHR culture consumed 4 L of water over 42 days, whereas the aeroponic culture required 40 L of water. HARs were exposed to an 18-hour photoperiod for 42 days, while HHRs can grow without light.

Although the two approaches to obtain hemp roots may not be fully equivalent to some extent, the comparison provides valuable insights into biomass productivity. Indeed, the HHR clone required several months of selection, whereas the plantlets germinated for 10 days before being cultivated in aeroponics. The nutrient requirements differ, while the whole plant relies primarily on photosynthesis for carbohydrate production, HHRs require adequate sucrose levels in the culture medium for growth. Finally, the HHRs were in a fully controlled and sterile environment, immersed in liquid media, and oxygenated thanks to spargers, whereas the HARs were exposed to the air and misted with a nutrient solution. Indeed, the HHRs demonstrated greater control and repeatability across batches, with a reduced risk of microbial contamination.

Aeroponic systems can be scaled-up in large greenhouses, and biomass can be partially harvested several times without sacrificing the whole plant, or the releasing of valuable compounds can be induced to enhance profitability [21]. Additionally, biomass production of the aeroponic root may be improved with a longer culture period. In a previous study, 238.7 ± 4.1 g FW or 15.8 ± 0.5 g DW of *C. sativa* root was obtained from 8 weeks of aeroponic culturing, still with variabilities in biomass amount per plant [12].

Hairy roots can also be scaled-up in larger bioreactors [31], biomass can be harvested by halting the culture, or the secretion of valuable molecules can be induced in the culture medium for better profitability [42]. Hairy roots could also reach higher amounts with optimized culture conditions and bioreactor designs [34,43]. For instance, 259 gFW/L and 15.3 g DW/L of *Artemisia annua* hairy roots were obtained in a 38-day culture in a 1.5 L working volume bubble column bioreactor [44]. This study is the first one to report biomass accumulation of hemp hairy roots in 1 L-bioreactors. Beyond biomass production, assessing the molecular content is essential for evaluating the efficiency of each technique.

### 2.2. Global Chemical Profiling of Hemp Hairy Roots (HHRs) by NMR and LC/MS

**Figure 2:**
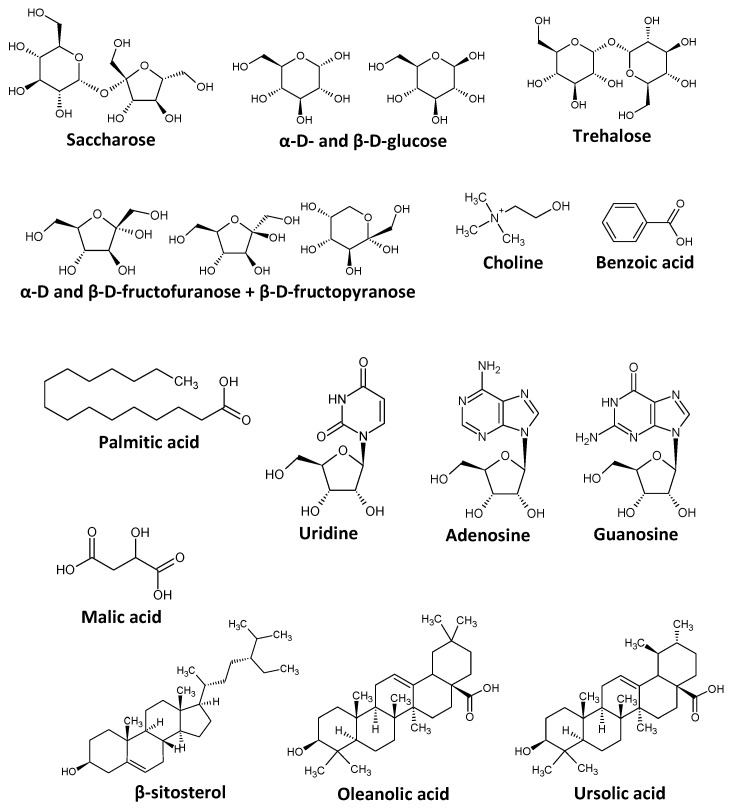
Chemical structures of the prevailing molecules unambiguously identified by NMR in HHR-UP and HHR-LOW.

A global chemical profiling performed on the HHR biomass to verify if its composition was similar to *C. sativa* roots composition, which has already been reported in the literature. NMR and LC/MS analysis were performed on dry biomass to characterize the chemical components of the HHRs. The total extraction yield was 37.5%, including 68 mg of HHR-UP and 453 mg of HHR-LOW, starting from 1390 mg of dry HHR biomass. The two extracts were analyzed using 1D and 2D NMR to determine the chemical classes and the major metabolites of the HHRs obtained in our cultivation conditions. Rigorous scrutinization of the whole NMR dataset (1H, 13C, HSQC, HMBC, and COSY spectra) led to the unambiguous identification of 14 metabolites predominating the composition of the two fractions (Figure 2). The major constituents of the most-polar extract HHR-LOW were monosaccharides, including glucose and fructose; disaccharides, including saccharose and trehalose; nucleosides, including uridine, adenosine, and guanosine; and choline and malic acid. These compounds were also detected in the less-polar extract HHR-UP, with a range of other metabolites of lower polarity, including palmitic acid, benzoic acid, β-sitosterol, and two acidic triterpenes, namely, oleanolic acid and ursolic acid. As the culture media of the HHRs was rich in saccharose, its composition exhibited many saccharides. The presence of triterpenes sterols and choline is consistent with the literature [6,11,38].

Significant NMR signals of phenolic compounds and lignanamides with tyramine moieties were also detected. However, these minor metabolites showed low concentrations, and therefore, it was difficult to fully resolve their chemical structures using NMR due to the very low signal intensity.

**Figure 3:**
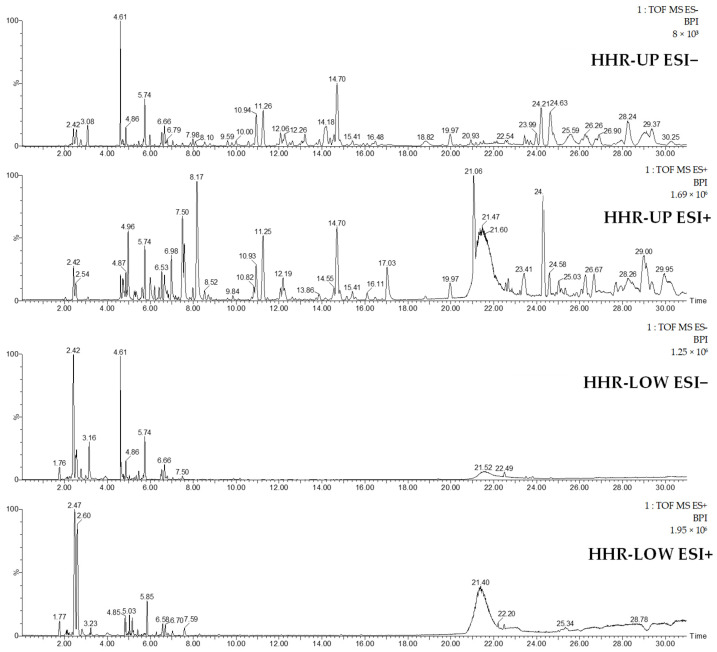
LC/MS BPI chromatogram (ESI+ and ESI−) of the extracts HHR-UP and HHR-LOW.

The two extracts were further analyzed using high-resolution LC/MS to reinforce the identification process. Acquisition was performed in both the positive and negative ionization modes. The resulting BPI chromatograms are given in Figure 3.

The LC/MS chromatograms showed that HHR-UP presented a more complex and diverse chemical profile, while HHR-LOW showed a less complex profile, with most of the pics regrouping at the left of the chromatogram. ESI+ and ESI− presented a different polarity, showing an extensive range of metabolites. The annotation of the chromatograms is reported in Table 2.

LC/MS revealed the potential presence of additional compounds not observed by NMR, such as opines, nucleosides, peptides, alkaloids (including cannabisativine), and other lipidic constituents. More particularly, HHR-UP likely shows the presence of lignanamides, including *N*-coumaroyltyramine glucoside, *N*-feruloyltyramine glucoside, *N*-caffeoyltyramine, *N*-coumaroyltyramine, and *N*-feruloyltyramine, as well as cannabisin D, cannabisin G, cannabisin F, grossamide, or its isomer.

Lignanamides are lignan compounds with amide groups, presenting hydroxycinnamic acid structures. They were first discovered in barley [45] and formed a group of more than 80 compounds further identified in about twenty plants [46]. However, this metabolite family remains relatively uncommon and distinctive. Thyramine derivates such as *N*-feruloyltyramine and *N*-coumaroyltyramine have already been identified in hemp roots [18,19]. However, *N*-caffeolytyramine seems to be newly identified in hemp roots, as was described only in hemp seeds [47].

**Figure 4:**
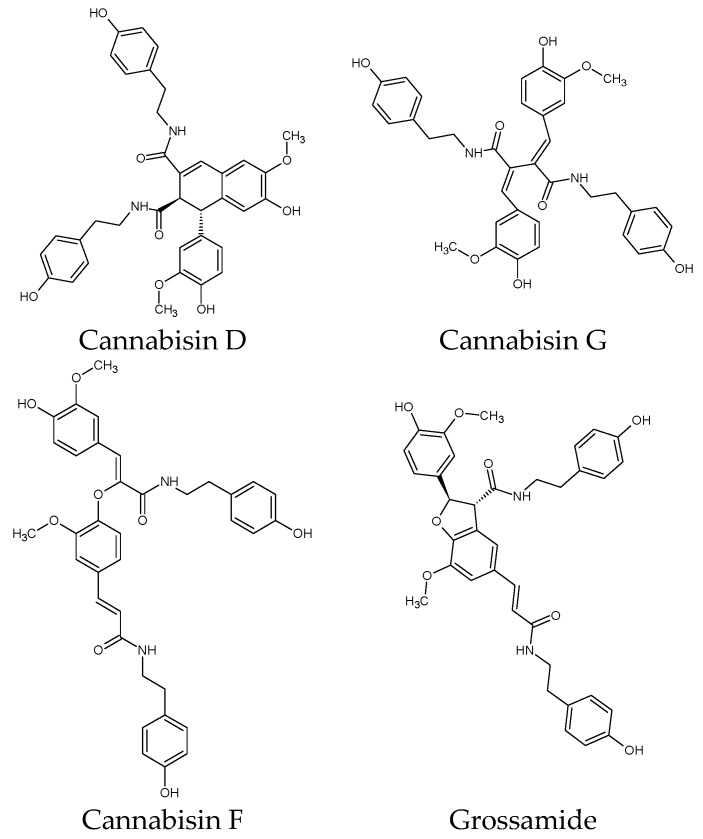
Chemical structures of cannabisins.

Additionally, to the best of our knowledge, this is the first description of cannabisin in hemp root tissue. Cannabisins have only been identified in cannabis fruits and seeds [46,48,49,50,51], as well as cannabisin D in *C. sativa* leaves [52]. Moreover, other cannabisin structures were identified in hemp seeds, such as cannabisin A, B, C, and E [50,53,54], as well as cannabisins M, N, and O [51]. Moreover, other cannabisin-like structures were identified in other plants, such as grossamide K and erythro-canabisin H in *Hibiscus cannabinus* [55], cannabisin K and L in *Hyoscyamus niger* seeds, and others [46]. The chemical structures of cannabisins D, G, and F, as well as grossamide, are given in Figure 4.

Additionally, a percentage of 38% of molecules was designated as Not Assigned, because no relevant correspondence was found within the databases. This represents an additional resource that will become accessible once we have made further progress in our understanding of natural resources. Indeed only a limited part of phyto-molecules is known to date (estimated between 6% and 15%) [56,57,58].

### 2.3. Quantification of Cannabisins and Triterpenes in HHR and HAR

**Figure 5:**
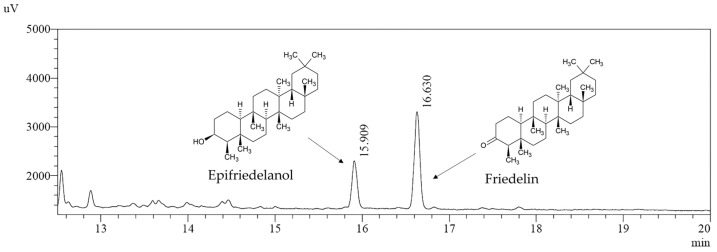
Chromatograms of friedelin and epifriedelanol quantification in HHR DMSO extract by GC-FID and their chemical structures.

To reinforce the characterization of the HHRs, we performed GC-FID analysis with friedelin and epifriedelanol standards. Indeed, these compounds are frequently reported in the literature about *C. sativa* roots but were not detected by NMR and LC/MS, likely due to their higher non-polarity that may not allow their extraction using the previous method. One more, acidic triterpenes (ursolic and oleanolic acid) were identified probably because of their greater polarity. The GC-FID analysis revealed the presence of friedelin and epifriedelanol, and this method proved to be complementary to LC/MS by identifying these more non-polar molecules that were not detected by the latter method. Chromatograms and the chemical structures of friedelin and epifriedelanol are given in Figure 5. Additional GC-FID chromatograms of standards are available in Figure S1.

To confirm cannabisin presence in HHRs, we performed UHPLC-QToF analysis with cannabisin D, cannabisin F, and grossamide standards. Cannabisin G was not quantified, because its standard was not available. Our results confirmed the presence of cannabisin D in HHR. However, under the current analytical conditions, cannabisin F and grossamide were co-eluted, which did not allow their separation and individual quantification. Indeed, the two molecule structures are close, and the signal was the same for both molecules using this method. As a result, cannabisins F is presented here as equivalent to grossamide.

**Figure 6:**
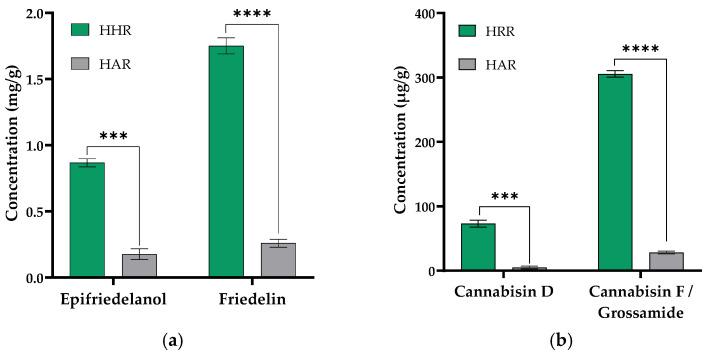
Quantification of triterpenes and cannabisins in HHRs and HARs. (**a**) Quantification of triterpenes from DMSO extracts of HHRs and HARs using GC-FID. Significantly higher amounts of triterpenes have been quantified in HHRs compared to HARs. Data are shown in mg/g. (**b**) Quantification of cannabisins from methanolic extracts of HHRs and HARs using UHPLC-QToF. Significantly higher amounts of cannabisins have been quantified in HHRs compared to HARs. Data are shown in µg/g. The values are displayed as the mean ± standard deviation of the biological triplicate (*n* = 3) and the technical triplicate (*n* = 3). Significant differences are indicated by asterisks: ***, *p* < 0.001; ****, *p* < 0.00001.

To further understand HHR metabolite production, we compared the accumulation of friedlin, epifriedelanol, cannabisin D, and cannabisin F equivalent to grossamide in the HHR and HAR extracts. Our results show that the HHRs were found to accumulate 0.86 ± 0.03 mg/g and 1.76 ± 0.05 mg/g of epifriedelanol and friedelin and 73.13 ± 4.52 µg/g and 305.67 ± 4.11 µg/g of cannabisin D and cannabisin F equivalent to grossamide. Only 0.17 ± 0.04 mg/g and 0.26 ± 0.03 mg/g of epifriedelanol and friedelin and 5.0 ± 1.63 µg/g and 28.33 ± 2.08 µg/g of cannabisin D and cannabisin F equivalent to grossamide were found to accumulate in HARs in average. This result supports that the HHRs accumulated significantly higher amounts of triterpenes and cannabisins than the HARs (Figure 6), in addition to achieve greater biomass in bioreactors. Additional UHPLC-QToF chromatograms of the quantification are available in Figure S2.

In several studies, hairy roots have shown to accumulate more metabolites than mother plants [35,59,60]. For instance, hemp hairy roots have shown a higher choline accumulation compared to untransformed control hemp roots [38]. More broadly, hairy roots transfected with *Rhizobium* have been shown to exhibit enhanced metabolic activity, and *rol* genes seem involved in this mechanism [61,62]. For instance, *Artemisia carvifolia* was transformed with *rol* B and *rol* C genes and revealed an increased level of flavonoid content by 1.9- to 6-fold and 1.6- to 4-fold, respectively [63].

To the best of our knowledge, this is the first report of cannabisin amounts detected in *C. sativa* or hemp roots. The use of hairy roots highlighted the presence of cannabisins, which were in very low concentrations in the HARs. This low concentration may account for the prior lack of identification of cannabisins in this plant part. Indeed, cannabisins has only been identified in *C. sativa* seeds and fruits [46,48,49,50,51] or leaves so far [52]. In hemp seed, cannabisin A, B, and M were quantified at 74.23 µg/mg, 55.52 µg/mg, and 10.33 µg/mg, respectively [46,64]. Compared to hempseeds, HHR accumulates more than 2700-fold more cannabisins, with a total quantification of 378 µg/g. To our knowledge, no data are available on the quantification of other cannabisins.

Several studies have reported the accumulation of epifriedelanol and friedelin in hemp roots cultivated in soil [6], in aeroponic hemp roots [25], and in hemp hairy roots [39,40]. These results reveal that the HHRs approach was more efficient to accumulate triterpenes compared to other methods. They show a total accumulation of epidredelanol and friedelin reaching up to 2.62 mg/g, while the maximum reported amount in hemp soil roots was 2.27 mg/g [6], and in hemp aeroponic roots it was 0.51 mg/g [25]. Notably, the HHRs in our study showed higher triterpenes content than previous studies on hemp hairy roots. In basal conditions, previous studies showed epifredelanol and friedelin amounts of 0.39 mg/g and 0.49 mg/g at 6 days of culture in Erlenmeyer [39] and 0.57 mg/g and 0.96 mg/g at 28 days of culture in Erlenmeyer [40]. The difference in accumulation between different hemp hairy root clones may be due to the selected clone, the culture conditions, and the *Rhizobium* infection transferring its T-DNA in random and multiple-copy insertion into the plant genome. Some insertions may be more favorable for metabolite production than others [65,66]; indeed, each HHR clone is unique.

Otherwise, in our experimental conditions, the HARs demonstrated lower amounts of epifriedelanol and friedelin than hemp root cultivated in soil (0.62 ± 0.29 mg/g and 1.09 ± 0.26 mg/g) [6]. The exposure of soil-grown plants to micro-organisms may stimulate the production of these metabolites as a defense mechanism in soil-grown roots [67]. However, the epifredelanol and friedelin amounts in our HAR cultures are comparable to those of previous hemp aeroponic roots (0.23 mg/g and 0.28 mg/g) [25]. Additionally, the triterpenes content in hemp root was found to vary with several factors. In Kornpointner et al., 2021, three hemp chemovars were used that showed relative variations of friedelin content in hemp soil roots (0.4 to 0.7 mg/g), where the Felina 32 variety presented the highest content. Harvest time and drying lead to variation, showing different contents of epifredelanol (0.059 mg/g to 0.20 mg/g) and friedelin (0.10 mg/g to 0.43 mg/g). The warm drying method showed the lowest amounts, and the best results were observed for 3-month-old samples. Finally, the extraction methods showed variation in their epifredelanol contents (0.016 mg/g to 0.18 mg/g), and ethanolic extraction showed the maximum amount [11].

Additionally, the use of elicitors can further enhance triterpenes accumulation, especially the use of salicylic acid. In hemp aeroponic roots, the epifredelanol and friedelin contents slightly been increased by 1.7- and 1.1-fold using 25 µM of salicylic acid [12]. In the hemp hairy root cultured in a flask, the epifredelanol and friedelin content also increased by 1.4- and 1.95-fold (0.68 mg/g and 0.96 mg/g) using 75 µM of salicylic acid [39]. Still, our HHR culture in the bioreactor, without elicitation, showed 1.2- and 1.8-fold higher contents of epifriedelanol and friedelin than the previous studies (0.86 mg/g and 1.76 mg/g). Moreover, in Mahendran et al., 2024, a maximum accumulation of epifredelanol (5.018 mg/g) and friedelin (1.56 mg/g) was reached using 100 µM and 50 µM salicylic acid, respectively, after 96 h of treatment, corresponding to an increase of 5.22- and 2.88-fold compared to the untreated material [40]. In these two previous studies on hemp hairy root elicitation, methyl jasmonate [39,40], chitosan, and yeast extract [39] were also tested, but these were less efficient in triggering triterpene accumulation. More broadly, other elicitors such as jasmonic acid, acetylsalicylic acid, and pectin have also been studied to enhance the production of triterpenes in various hairy root models [68,69,70,71]. These elements opened way to even more increases in the triterpenes content of our HHR culture.

To the best of our knowledge, no previous studies reported elicitation to produce lignanamides, including cannabisins. However, lignanmides are part of lignans and should share common pathways and regulation mechanisms [46]. For instance, MeJA proved to regulate lignan-like compound accumulation in *Isatis indigotica* hairy roots [72]. Another study showed the enhancement of lariciresinol and pinoresinol by 14.8- and 8.7-fold in *Linum album* hairy roots using coniferaldehyde acid 2 mM [73]. Further research is warranted to explore the elicitation mechanisms of lignanamides.

### 2.4. Activity Assays

**Figure 7:**
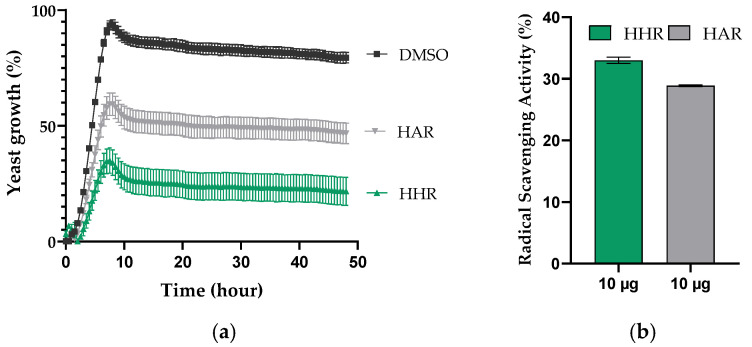
Antifungal and antioxidant activities of HHR and HAR non-polar extracts: (**a**) Inhibition of *Saccharomyces cerevisiae* growth over time by 200 µg of HHR and HAR non-polar extracts prepared at 100 mg/mL in DMSO with DMSO as a negative control. (**b**) Radical Scavenging Activity shows as gallic acid equivalent, showing assessment of HHR and HAR non-polar extract antioxidant activities at 10 µg in reaction to ABTS solution. The values are displayed as the mean ± standard deviation of the technical replicate (*n* = 3).

The activity assays were conducted on HHR and HAR non-polar extracts, prepared as HHR-UP, as the non-polar extract exhibited a higher chemical diversity than the polar extract. The potential antifungal and antioxidant activity (ABTS) of the HHR and HAR non-polar extracts are reported in Figure 7.

The HHR extract demonstrated higher activity than the HAR extract. A significant *S. cerevisaie* growth inhibition of 59% was observed for the HHR non-polar extract compared to a moderate inhibition of 34% for the HAR one. Both extracts showed moderate antioxidant activity. Slightly superior antioxidant activity was found in the HHR non-polar extract, showing 34% compared to 29% for the HAR extract. These first activity assays should be validated by further tests but suggest an improved activity of the HHR non-polar extract.

*C. sativa* roots extract has demonstrated antimicrobial and antioxidant properties. For instance, *C. sativa* roots extracted by subcritical CO_2_ demonstrated inhibitory effects against pathogens, including Staphylococcus aureus (involved in skin infections) and *Candida albicans* (involved in microbiome skin imbalance) [74]. Additionally, *C. sativa* ethanolic roots extract have demonstrated strong antioxidant activity using several assays, including the ABTS assay, Ferric Reducing Antioxidant Power assay (FRAP), and intracellular oxidation [11].

Concerning antimicrobial activity, it is well established that roots exhibit such properties due to their interaction with soil micro-organisms. This interaction promotes the development of antimicrobial compounds, enabling roots to counter or connect with these microbial communities [75,76,77]. However, the HHR extract coming from sterile in vitro culture showed surprisingly higher antifungal activity. The enhanced metabolism of the HHRs over the HARs could explain the increased antimicrobial activity of the HHR non-polar extracts.

The metabolite composition of the HHR non-polar extract suggests antioxidant and anti-inflammatory effects. Indeed, lignanamides displayed promising antioxidants and anti-inflammatory activities in several studies. For instance, *Lycium chinense* Miller roots were studied for their anti-inflammatory properties, revealing tyramine derivates (including *N*-coumaroylthyramine and *N*-feruoylthramine) as potential compounds for the inhibition of TNF-α and *N*-caffeoylthyramine as the main responsible compound for NF-κB inhibition in in vitro models [78]. *N*-caffeoyltyramine anti-inflammation properties were further confirmed on LPS-treated human primary monocytes with hempseed extract, showing a reduction in TNF-α and IL-6 gene expression and secretion [47]. Moreover, this compound demonstrated promising antioxidant activity from *Celtis* varieties [79,80]. Few studies can be found on cannabisin and suggest anti-inflammatory activities. For instance, hempseed extracts composed of cannabisin F and grossamide showed potent inhibition of IL-6 and TNF-α in LPS-induced BV2 microglia cells [81,82]. Additionally, cannabisin B from hempseed showed a potent antioxidant activity [83].

Triterpenes are also reported to be antioxidant and anti-inflammatory. Ursolic acid and oleanolic acid were identified as major compounds of the HHRs in our NMR and demonstrated those properties in several studies [84], showing significant reductions in IL-6 and TNF-α in mouse tissue, among other effects [85]. More specifically, *C. sativa* root extracts composed of friedelin, epifriedelanol β-sitosterol, and cannabisativine demonstrated potential anti-inflammation properties [8]. For instance, friedelin in hemp root extract was identified as the main active compound to inhibit IL-1β production in THP-1 cells, showing 78% activity [7]. These findings suggest a potential anti-inflammatory effect that could be assessed in further studies. 

## 3. Materials and Methods

### 3.1. Plant Materials

#### 3.1.1. Culture of Hemp Hairy Roots (HHR)

**Figure 8:**
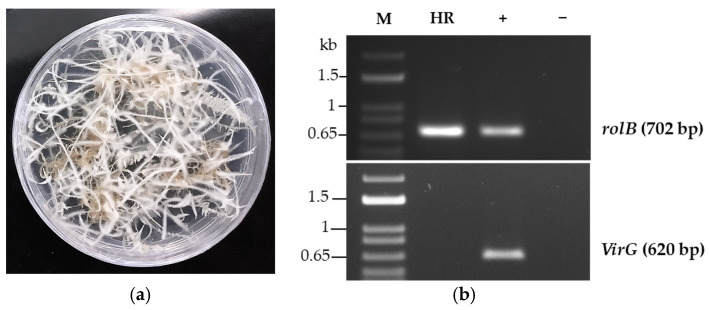
Establishment of hemp hairy root clones. (**a**) Selected hemp hairy root clone in solid culture media. (**b**) PCR analysis of HHR transformed with *R. rhizogenes* to verify the presence of *rol* B (T-DNA marker) and *Vir* G genes (*R. rhizogenes* marker). The DNA of *R. rhizogenes* was used as positive control (+) and water as negative control (−).

Hemp hairy root (HHR) clones of *C. sativa* were generated as described in the protocol of Wahby et al. [86]. Sterile two-week hemp plantlets (Hemp it) were transfected with wild-type *Rhizobium rhizogenes*. The most vigorously growing HHR clone was selected on Murashige and Skoog (MS) medium [87] and enriched in sucrose. *R. rhizobium* was eliminated using cefotaxime, which was removed at the end of the selection. Figure 8a shows the obtained HHR clone. The presence of *rol* B (T-DNA positive marker) and absence of *Vir G* (*R. rhizogenes* presence marker) were confirmed by Polymerase Chain Reaction (PCR) to validate the transfection of the T-DNA into the biomass (Figure 8b).

Selected HHR tissues were transferred to a liquid suspension medium composed of ½ Murashige and Skoog (MS), supplemented with 3% sucrose, and adjusted to pH 5.8. The cultures were maintained at room temperature, rotating at 80 rpm, and subcultured over 21 days in Erlenmeyer in dark. The precultures were further scaled up to 1-L oxygenated bioreactors and supported by stainless steel baskets. To test for reproducibility, we performed three independent experiments of HHR culture in a bioreactor (*n* = 3). After a total of 42 days of in liquid culture, the biomass was harvested, separated from the media, washed, and freeze-dried for chemical and activity analysis.

#### 3.1.2. Culture of Hemp Aeroponic Roots (HARs)

Hemp seeds (Hemp it) were germinated in mineral wool cubes pre-soaked in water at pH 5.8. Once the plantlets developed four leaves, they were transferred to an aeroponic system (CultiMate^®^, Berlin, Germany) in argil beads and fed with a nutrient solution (TriPart^®^, Fleurance, France). The system operated under an 18-h-light/6-h-dark-controlled photoperiod. The nutrient solution was replaced weekly, and Electrical Conductivity (EC) levels were progressively increased from 0.5 to 1.5 mS/cm, while pH was consistently maintained at 5.8 throughout the cultivation period. After 42 days of growth, the hemp aeroponic roots (HARs) were harvested, washed with distilled water, and freeze-dried for further analysis. Three independent plants were used for experiments (*n* = 3).

### 3.2. Global Chemical Profiling of Hemp Hairy Roots (HHRs) Through NMR and LC-MS

#### 3.2.1. Chemicals

Ethyl acetate, acetonitrile, and methanol (MeOH) were purchased from Carlo Erba (Val de Reuil, France). Acetic acid and formic acid were purchased from VWR (Radnor, PA, USA). Deionized water was used to prepare all aqueous solutions.

#### 3.2.2. Extract Preparation and Fractionation

A biphasic solvent system of ethyl acetate, acetonitrile, and water was prepared in 3/3/4 (*v*/*v*/*v*) proportions in a separating funnel. After solvent equilibration and decantation, the two immiscible liquid phases were separated, and 40 mL of both phases were added to 1390 mg of dry ground hairy roots. The mixture was placed in an ultrasonic bath for 1 h at room temperature and filtered. Then, the two liquid phases of the biphasic solvent were separated and evaporated to dryness with a rotary evaporator, resulting in two sub-extracts labeled HHR-UP for non-polar fraction and HHR-LOW for polar fraction.

#### 3.2.3. NMR Analysis and Identification of the Prevailing Molecules

An aliquot of HHR-UP or HHR-LOW (20 mg) was dissolved in 600 µL of DiMéthylSufOxide-*d*_6_ (DMSO) and analyzed by 1H, 13C, Heteronuclear Single Quantum Coherence (HSQC), Heteronuclear Multiple Bond Correlation (HMBC), and Correlation Spectroscopy (COSY) NMR at 298 K on a Bruker Avance AVIII-600 spectrometer (Bruker, Karlsruhe, Germany) equipped with a TCI cryoprobe. All spectra were manually phased and baseline corrected using the TOPSPIN 4.0.5 software from Bruker and calibrated on the central resonance of DMSO-*d*_6_ at 39.80 ppm. Structural elucidation of metabolites was performed by rigorous scrutinization of the 1D and 2D NMR datasets. All metabolites unambiguously identified using this NMR approach are presented in Figure 2.

#### 3.2.4. Liquid Chromatography/Mass Spectrometry (LC/MS) Analyses

The two extracts, HHR-UP and HHR-LOW, were also analyzed by high-resolution LC/MS in the positive and negative ion modes on an Acquity UPLC H-Class system (Waters, Manchester, UK) coupled to a Synapt G2-Si from Waters equipped with an electrospray (ESI) ion source. Both samples were prepared at 1 g/L in MeOH/H_2_O (1/1, *v*/*v*). The chromatographic column was a Uptisphere C-18 ODB 150 × 4.6 mm, 5 µm from Interchim (Montluçon, France), maintained at 35 °C. The mobile phase gradient started with 100% of solvent A (MilliQ water (Millipore, Burlington, MA, USA) + 0.1% formic acid) and 0% solvent B (acetonitrile + 0.1% formic acid), then increased to 26% solvent B in 4 min, to 65% solvent B from 4 to 18.5 min, to 100% solvent B from 18.5 to 18.7 min, maintained at 100% solvent B until 26 min, and recycled back to 100% solvent A for 5 min. The flow rate was 0.7 mL/min, and the sample injection volume was 5 µL. MS acquisition was performed within the scan range 50 < *m*/*z* < 2000. The capillary voltage was set at 3 kV, the desolvation temperature at 650 °C, the desolvation gas flow at 750 L/h, the source temperature at 150 °C, the cone voltage at 40 V, and the cone gas flow at 50 L/h. Data were processed using the MassLynx software V4.2 from Waters. All detected peaks were assigned to a molecular formula based on exact mass measurements, and a chemical structure was proposed for each formula based on the literature data. The resulting BPI chromatograms are given in Figure 3, and LC/MS data are summarized in Table 2.

### 3.3. Quantification of Specific Triterpenes and Cannabisins in HHRs and HARs

#### 3.3.1. GC-FID Quantification of Epifriedelanol and Friedelin

Epifriedelanol and friedelin standards were purchased from Merck (Darmstadt, Germany). The content of these molecules was investigated in dried samples of HHRs (*n* = 3) and HARs (*n* = 3). Dry samples of 300 mg were ground in powder, extraction was performed using 10 mL of DMSO, and the extract was further treated with ultrasound for 20 min.

The Gas Chromatography–Flame Ionization Detection (GC-FID, Shimadzu, Kyoto, Japan) analysis was performed using a system equipped with a Rxi-35Sil MS column (30 m length, 0.25 mm internal diameter, and 0.25 µm film thickness). The injector temperature was set to 250 °C, and the injection was carried out in split mode with a split ratio of 50:1. Helium was used as the carrier gas with a constant linear velocity of 41.0 cm/s, a column flow rate of 1.50 mL/min, and a total flow of 79.5 mL/min. The pressure was maintained at 189 kPa, and a 3.0 mL/min purge flow was applied. The oven temperature was initially held at 225 °C for 0.50 min, followed by a ramp of 10 °C/min to 325 °C held for 20 min. The detector temperature was maintained at 350 °C throughout the analysis. The flame ionization detector used helium as the makeup gas at a 30.0 mL/min flow rate, with hydrogen and air flow rates set to 40.0 mL/min and 400.0 mL/min, respectively. The total run time was 30.50 min, ensuring the separation and detection of a wide range of volatile organic compounds. For calibration, the concentration of analytes followed a linear model, ensuring that the signal response was proportional to the concentration of the compounds under investigation, allowing for accurate quantification. The analysis was conducted on biological triplicate for each biomass (*n* = 3) and in technical triplicates for each sample (*n* = 3), and the results are presented in Figure 5 and Figure 6.

#### 3.3.2. UHPLC-QToF Quantification of Cannabisins

Grossamide, cannabisin F, and cannabisin D standard were purchased from ChemFaces (Wuhan, China). The contents of these molecules were investigated in dried samples of HHRs (*n* = 3) and HARs (*n* = 3). A mechanical and methanolic extraction were performed using a FastPrep bead beater (Irvine, CA, USA, two cycles of 30 s at 6.5 m/s) and 5 min of ultrasound. An amount of 10 mg of sample was milled using glass beads of 0.5–3 mm in 10 mL of methanol. 

An Ultra-High Performance Liquid Chromatography (UHPLC, Bruker) with a Quadrupole Time-of-Flight (TIMS-ToF, Bruker) was developed to analyze grossamide and cannabisins contents. The chromatographic separation was performed using an Acquity UPLC HSS T3 column (2.1 × 150 mm, 1.8 µm) from Waters. The column was maintained at 35 °C. The mobile phase combines eluent A: Milli-Q water and eluent B: methanol. Elution was achieved at a flow rate of 0.3 mL/min in gradient mode starting at 20% B for 1 min; the proportion of B was gradually increased to 100% in 19 min and held at 100% for 10 min. The gradient was then returned to its initial condition and left to stabilize for 10 min. The total duration of the analysis was 40 min. The injection volume was 1 µL. Mass spectrometry data were acquired at 10 Hz with a 20–1300 *m*/*z* range in auto MS/MS mode. ESI source was set up with a capillary voltage of 4 kV, a desolvation temperature of 220 °C, a gas flow of 10 L/min, and a nebulizer pressure at 2.2 Bar. Data were processed using Target Analysis Screening Quantification (TASQ) 2023b from Bruker. A stock solution was prepared from standards of cannabisin D, cannabisin F and grossamide at a concentration of 100 mg/L in methanol, respectively. This was used to create a calibration range from 2 to 2000 µg/L with 8 range points. The analysis was conducted on biological triplicate for each biomass (*n* = 3) and in technical triplicates for each sample (*n* = 3) in negative mode (ESI−), and the results are presented in Figure 6. 

#### 3.3.3. Statistical Analysis

Statistical analysis was performed with Prism 10 (Graphpad, La Jolla, CA, USA) software. Multiple unpaired t-tests were used to analyze the triterpene and cannabisin quantification. Significant differences are indicated by asterisks: *, *p* < 0.05; **, *p* < 0.01; ***, *p* < 0.001; ****, *p* < 0.00001; ns, non-significant difference.

### 3.4. Activity Assays

#### 3.4.1. Antifungal Assay

The inhibitory capacity against the unicellular fungus *Saccharomyces cerevisiae* growth was evaluated for HHR and HAR non-polar extracts prepared as HHR-UP (*n* = 1). The dry extracts were solubilized in DMSO to reach a final 100 mg/mL concentration. *S. cerevisiae* was cultivated under optimal growth conditions in Yeast Peptone Dextrose (YPD) medium at 30 °C. The study was performed in a 96-well plate; 198 µL of *S. cerevisiae* concentrated at 1 mg/mL and 2 µL of extract preparations were added in each well, corresponding to 200 µg of dry extracts. The growth of *S. cerevisiae* was measured by Optical Density (OD) of 600 nm for 48 h. The experiment was performed in technical triplicate with DMSO as a negative control.

#### 3.4.2. Antioxydant Assay (ABTS)

The radical scavenging activity was assessed for HHR and HAR non-polar extracts prepared as HHR-UP (*n* = 1). The dry extracts were solubilized in DMSO to reach a final 5 mg/mL concentration. A standard curve of gallic acid was generated and used as the oxidative agent in reaction with an ABTS (2,2′-azinobis [3-ethylbenzothiazoline-6-sulfonic acid]) solution, where the color intensity varies with the antioxidant activity. The study was performed in a 96-well plate; 198 µL of ABTS solution and 2 µL of extract preparations were added in each well, corresponding to 10 µg of dry extracts. Absorbance was monitored at 734 nm at room temperature. The experiment was performed in technical triplicate with DMSO as a negative control. The ABTS activity was determined using the formula:

*ABTS*, *Radical Scavenging Activity* (%) = [(*A*_0_ − *A_s_*)/*A*_0_] × 100

where *A*_0_ is the absorbance value of the negative control, and *A_s_* is the absorbance of the sample tested at 30 min.

## 4. Conclusions

In this study, we successfully developed and chemically profiled a hemp hairy root culture to elucidate its phytochemical composition and production, offering a comparative analysis with aeroponic hemp roots. While previous studies have investigated the metabolic content of hemp hairy roots in flask, focusing on compounds such as atropine, choline [38], and triterpenoids [39,40]. This research represents the first global phytochemical profiling of hemp hairy roots cultivated in a 1 L-bioreactor, highlighting the presence of cannabisins. LC/MS analysis uncovered a diverse array of bioactive compounds, including acidic triterpenes (notably oleanolic acid and ursolic acid), sterols (β-sitosterol), phenolic acids, fatty acids (both saturated and unsaturated), nucleosides (uridine, guanosine, and adenosine), and various tyramine derivatives. Notably, we identified cannabisins in hemp hairy roots, being the first report of these molecules in this plant part. Additionally, we found that hemp hairy roots accumulate significantly higher levels of cannabisins and triterpenes than other cultivation methods. Preliminary assessments also indicated that the extract exhibits antioxidant and antifungal activities, suggesting hemp hairy roots could be used as a promising source of plant-based metabolites. This study highlights the unique phytochemical profile of hemp hairy roots and underscores their potential for various applications. The advantages offered by hairy root cultures, such as improved productivity of biomass and metabolites, better reliability due to in vitro controlled culture and genetic consistency, and water- and energy-saving potential, make them a promising avenue for further exploration and utilization in industrial and medicinal contexts. Future research will aim to expand our understanding of the bioactive compounds within hemp hairy roots and their potential applications in various fields.

## Figures and Tables

**Table 1:** Harvested biomass of HHRs and HARs after 42 days of cultivation. Data indicate the biomass quantity in FW and DW in grams (g) presented as means with standard deviations for three biological replicates.

Culture System	FW (g)	DW (g)
**HHR**	57.31 ± 0.33	3.18 ± 0.08
**HAR**	27.08 ± 7.82	1.07 ± 0.32

**Table 2:** Summary of LC/MS data annotations.

LC Retention Time (min)	Observed *m*/*z*	Elemental Composition	Δppm	Annotation
**HHR-UP**				
2.42	311.1455 [M+H]^+^	C_11_H_23_N_2_O_8_	0.3	Glucopine, Mannopine, or isomer
	309.1294 [M−H]^−^	C_11_H_21_N_2_O_8_	−1.3	
2.54	312.1295 [M+H]^+^	C_11_H_22_NO_9_	0.0	Mannopinic acid or isomer
2.55	195.0500 [M−H]^−^	C_6_H_11_O_7_	−2.6	Gluconic acid or isomer
3.09	341.1085 [M−H]^−^	C_12_H_21_O_11_	0.3	Saccharose *
4.61	294.1194 [M+H]^+^	C_11_H_20_NO_8_	1.0	Not Assigned
	292.1029 [M−H]^−^	C_11_H_18_NO_8_	−1.0	
4.73	243.0618 [M−H]^−^	C_9_H_11_N_2_O_6_	0.4	Uridine *
4.74	268.1045 [M+H]^+^	C_10_H_14_N_5_O_4_	−0.1	Adenosine *
4.82	282.0836 [M−H]^−^	C_10_H_12_N_5_O	−0.7	Guanosine *
4.96	180.1389 [M+H]^+^	C_11_H_18_NO	0.6	Not Assigned
5.25	253.1188 [M+H]^+^	C_12_H_17_N_2_O_4_	0.0	Dipeptide Phe-Ser or isomer
5.28	144.0485 [M+H]^+^	?	-	Not Assigned
5.36	270.2542 [M+H]^+^	?	-	Not Assigned
5.61	380.2914 [M+H]^+^	C_21_H_38_N_3_O_3_	0.3	Not Assigned
5.74	267.1345 [M+H]^+^	C_13_H_19_N_2_O_4_	0.0	Dipeptide Thr-Phe or isomer
	265.1189 [M−H]^−^	C_13_H_17_N_2_O_4_	0.4	
5.98	299.0770 [M−H]^−^	C_13_H_15_O_8_	1.0	Hydroxybenzoyl glucose
5.98	382.3071 [M+H]^+^	C_21_H_40_N_3_O_3_	0.3	Cannabisativine
6.20	188.0713 [M+H]^+^	C_11_H_10_NO_2_	0.5	Not Assigned
6.41	284.2338 [M+H]^+^	C_15_H_30_N_3_O_2_	0.0	Not Assigned
6.54	233.1504 [M+H]^+^	C_10_H_21_N_2_O_4_	1.3	Dipeptide Thr-Leu or isomer
	231.1345 [M−H]^−^	C_10_H_19_N_2_O_4_	0.0	
6.66	217.1554 [M+H]^+^	C_10_H_21_N_2_O_3_	0.3	Dipeptide Val-Val or isomer
	215.1400 [M−H]^−^	C_10_H_19_N_2_O_3_	1.9	
6.79	215.1397 [M−H]^−^	C_10_H_19_N_2_O_3_	0.5	Dipeptide Val-Val or isomer
6.98	266.2232 [M+H]^+^	?	-	Not Assigned
7.05	249.1244 [M−H]^−^	C_13_H_17_N_2_O_3_	2.0	Not Assigned
7.22	467.1552 [M−H]^−^	C_22_H_27_O_11_	−0.2	Not Assigned
7.50	236.0556 [M−H]^−^	C_11_H_10_NO_5_	−1.3	Not Assigned
7.50	268.2386 [M+H]^+^	C_15_H_30_N_3_O	−1.1	Not Assigned
7.59	326.2445 [M+H]^+^	C_17_H_32_N_3_O_3_	0.3	Not Assigned
7.87	444.1658 [M−H]^−^	C_23_H_26_NO_8_	0.0	*N*-Coumaroyltyramine glucoside
	282.1129 [M−H−Glu]^−^	C_17_H_16_NO_3_	−0.4	*N*-Coumaroyltyramine fragment
7.98	476.1922 [M+H]^+^	C_24_H_30_NO_9_	0.2	*N*-Feruloyltyramine glucoside
	474.1771 [M−H]^−^	C_24_H_28_NO_9_	1.5	*N*-Feruloyltyramine glucoside
	312.1239 [M−H−Glu]^−^	C_18_H_18_NO_4_	1.0	*N*-Feruloyltyramine fragment
8.10	474.1762 [M−H]^−^	C_24_H_28_NO_9_	−0.4	*N*-Feruloyltyramine glucoside isomer2
8.20	328.2602 [M+H]^+^	C_17_H_34_N_3_O_3_	0.6	Not Assigned
8.53	488.1920 [M−H]^−^	C_25_H_30_NO_9_	−0.2	Not Assigned
8.78	121.0290 [M−H]^−^	C_7_H_5_O_2_	0.0	Benzoic acid *
9.59	298.1076 [M−H]^−^	C_17_H_16_NO_4_	−1.0	*N*-Caffeoyltyramine
9.80	453.2337 [M−H]^−^	C_20_H_37_O_11_	0.2	Not Assigned
10.00	187.0966 [M−H]^−^	C_9_H_15_O_4_	−2.1	Azelaic acid
10.57	423.2235 [M−H]^−^	C_19_H_35_O_10_	1.2	Not Assigned
10.82	325.2277 [M+H]^+^	C_21_H_29_N_2_O	−0.9	Not Assigned
10.94	284.1288 [M+H]^+^	C_17_H_18_NO_3_	0.4	*N*-Coumaroyltyramine
	282.1132 [M−H]^−^	C_17_H_16_NO_3_	0.7	
11.22	314.1393 [M+H]^+^	C_18_H_20_NO_4_	0.3	*N*-Feruloyltyramine
	312.1241 [M−H]^−^	C_18_H_18_NO_4_	1.0	
12.06	625.2550 [M+H]^+^	C_36_H_37_N_2_O_8_	0.0	Cannabisin D or G or F or Grossamide or isomer
	623.2396 [M−H]^−^	C_36_H_35_N_2_O_8_	0.5	
12.19	328.1548 [M+H]^+^	C_19_H_22_NO_4_	−0.3	Not Assigned
12.27	625.2550 [M+H]^+^	C_36_H_37_N_2_O_8_	0.0	Cannabisin D or G or F or Grossamide or isomer
	623.2401 [M−H]^−^	C_36_H_35_N_2_O_8_	1.3	
13.21	327.2178 [M−H]^−^	C_18_H_31_O_5_	2.1	C18 fatty acid derivative
14.18	329.2334 [M−H]^−^	C_18_H_33_O_5_	1.8	C18 fatty acid derivative
14.55	565.2342 [M+H]^+^	C_34_H_33_N_2_O_6_	0.5	Not Assigned
14.70	625.2556 [M+H]^+^	C_36_H_37_N_2_O_8_	1.0	Cannabisin D or G or F or Grossamide or isomer
	623.2394 [M−H]^−^	C_36_H_35_N_2_O_8_	0.3	
15.41	639.2704 [M+H]^+^	C_37_H_39_N_2_O_8_	−0.3	Not Assigned
	637.2552 [M−H]^−^	C_37_H_37_N_2_O_8_	0.3	
16.11	316.2852 [M+H]^+^	C_18_H_38_NO_3_	0.0	Sphingosine derivative
16.49	934.3574 [M−H]^−^	?	-	Not Assigned
17.03	318.3006 [M+H]^+^	C_18_H_40_NO_3_	−0.6	Sphingosine derivative
18.82	476.2780 [M−H]^−^	?	-	Not Assigned
19.99	603.3898 [M+H]^+^	C_35_H_55_O_8_	0.2	Not Assigned
	601.3741 [M−H]^−^	C_35_H_53_O_8_	0.2	
24.21	471.3109 [M−H]^−^	C_29_H_43_O_5_	−0.2	Not Assigned
24.34	481.2936 [M+H]^+^	C_30_H_41_O_5_	−3.7	Not Assigned
24.63	271.2271 [M−H]^−^	C_16_H_31_O_3_	−0.7	Palmitic acid **
25.59	355.3218 [M−H]^−^	C_22_H_43_O_3_	1.7	Hydroxydocosanoic acid
26.26	455.3532 [M−H]^−^	C_30_H_47_O_3_	1.5	Triterpene (oleanolic or ursolic or isomer)
26.90	299.2593 [M−H]^−^	C_18_H_35_O_3_	2.3	C18 fatty acid derivative
28.22	605.4058 [M−H]^−^	C_36_H_53_N_4_O_4_	−1.5	Not Assigned
**HHR-LOW**				
2.42	311.1455 [M+H]^+^	C_11_H_23_N_2_O_8_	0.3	Glucopine, Mannopine, or isomer
	309.1294 [M−H]^−^	C_11_H_21_N_2_O_8_	−1.3	
2.54	312.1295 [M+H]^+^	C_11_H_22_NO_9_	0.0	Mannopinic acid or isomer
2.55	195.0500 [M−H]^−^	C_6_H_11_O_7_	−2.6	Gluconic acid or isomer
2.77	404.1041 [M−H]^−^	C_12_H_22_NO_14_	0.2	Not Assigned
3.09	341.1085 [M−H]^−^	C_12_H_21_O_11_	0.3	Saccharose *
3.18	253.0926 [M−H]^−^	C_9_H_17_O_8_	1.2	Glycerol hexoside
3.91	606.0735 [M−H]^−^	C_25_H_20_NO_17_	0.7	Not Assigned
4.61	294.1194 [M+H]^+^	C_11_H_20_NO_8_	1.0	Not Assigned
	292.1029 [M−H]^−^	C_11_H_18_NO_8_	−1.0	
4.82	282.0836 [M−H]^−^	C_10_H_12_N_5_O_5_	−0.7	Guanosine *
5.74	267.1345 [M+H]^+^	C_13_H_19_N_2_O_4_	0.0	Dipeptide Thr-Phe or isomer
	265.1189 [M−H]^−^	C_13_H_17_N_2_O_4_	0.4	
6.54	233.1504 [M+H]^+^	C_10_H_19_N_2_O_4_	1.3	Dipeptide Thr-Leu or isomer
	231.1345 [M−H]^−^		0.0	
6.66	217.1554 [M+H]^+^	C_10_H_21_N_2_O_3_	0.3	Dipeptide Val-Val or isomer
	215.1400 [M−H]^−^	C_10_H_19_N_2_O_3_	1.9	
6.98	266.2232 [M+H]+	?	-	Not Assigned
7.50	236.0556 [M−H]^−^	C_11_H_10_NO_5_	−1.3	Not Assigned
7.50	268.2386 [M+H]^+^	C_15_H_30_N_3_O	−1.1	Not Assigned
8.20	328.2602 [M+H]^+^	C_17_H_34_N_3_O_3_	0.6	Not Assigned

* Also identified by NMR ** confirmed with an analytical standard.

## Data Availability

The dataset is available on request from the authors.

## Supplementary Materials

The following supporting information can be downloaded at https://www.mdpi.com/article/10.3390/molecules29235792/s1: Figure S1. GC-FID chromatograms of standards epifredelanol and friedelin. Figure S2. UHPLC-QToF chromatograms of cannabisin D, cannabisin F, and grossamide quantification.
