# Supplementary material for: Deciphering the Phytochemical Potential of Hemp Hairy Roots: A Promising Source of Cannabisins and Triterpenes as Bioactive Compounds
Source: Molecules. 2024 Dec 7;29(23):5792. doi: 10.3390/molecules29235792 (PMC11643499; doi:10.3390/molecules29235792)
Supplement: Supplementary file 1 [file molecules-29-05792-s001.zip › molecules-3263343-supplementary.pdf]

Supplementary Materials.

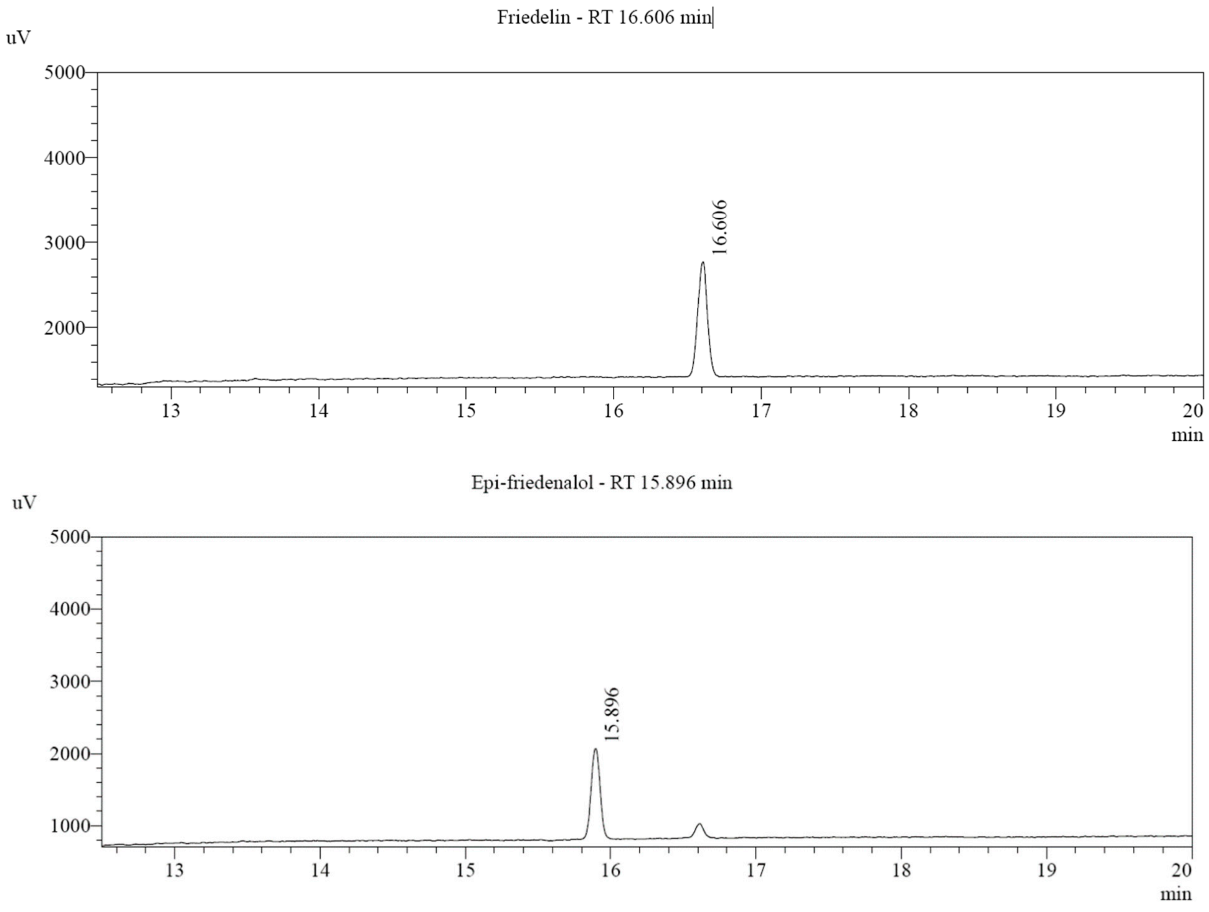

Figure S1. GC-FID chromatograms of standards friedelin and epifriedelanol

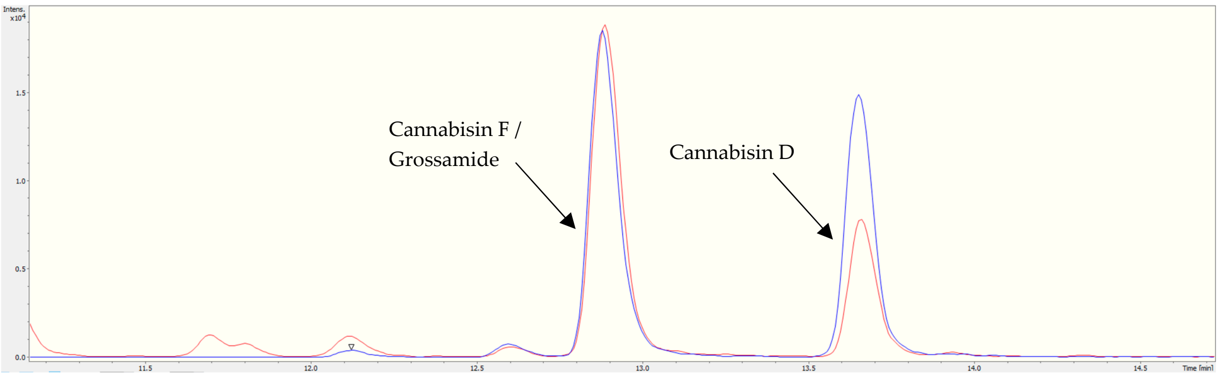

Figure S2. UHPLC-QToF chromatograms of cannabisin D, cannabisin F and grossamide quantification. Blue: standard of mixed cannabisin D, F and grossamide at 2 mg/L. Red: HHR sample. The peak at 12.9 min corresponds to cannabisin F and grossamide while the peak at 13.6 min is cannabin D.
